# Interference by Modern Smartphones and Accessories with Cardiac Pacemakers and Defibrillators

**DOI:** 10.1007/s11886-022-01653-0

**Published:** 2022-01-27

**Authors:** Fahd Nadeem, Cao Thach Tran, Estelle Torbey, Daniel Philbin, Carlos Morales, Michael Wu

**Affiliations:** Division of Cardiology, Department of Medicine, Lifespan Cardiovascular Institute and Brown University, Providence, RI USA

**Keywords:** Pacemaker, Implantable cardioverter/defibrillator, Magnet, Hall-effect sensor, Reed switch, Smartphone

## Abstract

**Purpose of Review:**

The risk of cardiac implantable electronic device (CIED) interference from cell phones was previously thought to be low based on older studies. Current generation of smartphones have incorporated more magnets for optimization of wireless charging, attachment of accessories, and convenience functionalities. These magnets have the potential to cause CIEDs to inadvertently revert into magnet mode. The purpose of this review is to summarize recent findings on smartphones and their accessories causing interference on CIEDs.

**Recent Findings:**

Recent reports have demonstrated that the iPhone 12 series and accessories have the capability to cause CIED magnetic interference.

**Summary:**

Current generation of smartphones, smartwatches, wireless headphones, and accessories have the potential to cause CIEDs to revert into magnet mode in both in vivo and ex vivo experiments. The risk of a clinically significant event is unlikely as long as the Food and Drug Administration (FDA) recommendations are followed; keeping smartphones and accessories at least six inches away from CIEDs.

## Introduction

Smartphone use has increased significantly over the last decade and it is now estimated that 85% of Americans currently own a smartphone [1]. At the same time, there are over 225,000 pacemakers and 130,000 implantable cardioverter defibrillators (ICDs) implanted in the USA annually [2]. Smartphones have the capacity to interfere with cardiac implantable electronic devices (CIEDs) in multiple ways. They can cause electromagnetic interference (EMI) from the emission of radiofrequency (RF) energy needed for cellular communication or they can induce magnet response from static magnet fields present in the cell phone components. Traditionally, EMI from cellphone RF energy was more of a concern leading to a series of studies in the 1990s and 2000s that found the overall risk to be low [3–10]. EMI from static magnetic fields causing CIEDs to revert into magnet mode was of little concern as previous generation of cell phones did not contain strong magnets. The current generation of smartphones have incorporated more magnets for optimization of wireless inductive charging, attachment of accessories, and keeping flexible display flip phones folded. Recent reports have demonstrated that these magnets are able to generate static magnetic fields strong enough to induce magnet mode on CIEDs when in close contact. We present a review of the data and the potential clinical impact of smartphone/accessories and CIED interactions.

## Cellular Phone RF Energy and CIED Interactions

The potential for cellphone interference from emitted RF energy became an area of interest in the 1990s. The largest study was performed by Hayes et al. and investigated EMI in 980 patients with CIEDs. In each of the patients, the authors tested 5 different cellphones and conducted a total of 5625 tests for EMI. The incidence of clinically significant EMI was 6.6% and EMI only occurred when the cellular phone was held directly over the pacemaker. There were no cases of clinically significant EMI occurring when it was held at the normal position over the ear [3]. These findings were corroborated by other studies carried out in the 1990s all of which suggested that the risk of EMI was not insignificant [3–5]. This led to the widely adapted FDA recommendation that cell phones should be maintained at a distance of six inches away from CIEDs [11].

Subsequent generation of cellphones switched from global system for mobile communication (GSM) to universal mobile telecommunication system (UMTS) and long-term evolution (LTE) for wireless communication. When compared to GSM, the newer LTE and UMTS have different signal properties and lower maximum power emission which have lower potential for CIED interference [6]. CIED manufacturers also adapted and started incorporating sense-amplifier filters, feedthrough capacitor filters, and noise reduction algorithms to help reduce the risk of EMI from cellular phones [7]. This led to a second series of studies carried out in the 2000s. Lennerz et al. tested the Samsung Galaxy S3, Nokia Lumia, and HTC One XL for CIED interference on 307 patients. Only one patient’s CIED was affected with pacing inhibition (< 2 s ventricular pause) and premature ventricular pacing which was not clinically significant [8]. Burri et al. studied 63 patients with CIEDs represented from all five major device manufacturers for EMI from Samsung Galaxy S4 and Apple iPhone 6 smartphones. They tested during phone standby mode, phone ringing, or during a call with the phones placed directly over the generator and in the left parasternal region. They found no cases of EMI or magnet interference in a total of 882 tests [6]. In another study of 148 patients, the iPhone 6 only caused a single case of EMI in which there was undersensing leading to symptomatic ventricular pacing [9]. In a study by Ismail et al., cell phones on the UMTS wireless communication network did not demonstrate any cases of EMI in 100 patients tested [10]. The cumulative results of the major studies can be seen in Table 1. The overall risk of EMI from RF energy emission used for cell phone communication appears to be significantly lower when compared to the initial studies carried out in the 1990s. This led to some researchers challenging the FDA recommendations of keeping cell phones six inches away from CIEDs.

**Table 1:** Summary of cellular phone RF energy and CIED interactions

**Author**	**Date of publication**	**Number of patients**	**Wireless network**	**Incidence of CIED interference**
Barbaro	1995	101	GSM	25.7%
Hayes	1997	980	NADC, TDMA, PCS, CDMA	6.6%
Altamura	1997	200	GSM, TACS	21.5% (GSM), 17.5% (TACS)
Hekmet	2004	100	GSM	2%
Ismail	2010	100	UMTS	0
Burri	2016	63	Not stated	0%
Lennerz	2017	307	GSM, UMTS, LTE	0.3%
Lacour	2020	148	Not stated	0.7%

*CDMA* code division multiple access, *GSM* global system for mobile communications, *LTE* long-term evolution, *NADC* North American digital cellular, *PCS* personal communications service, *TACS* total access communication system, *TDMA* time-division multiple access, *UMTS* universal mobile telecommunications service

Although the risk of EMI from cellular phone RF energy emission on modern day devices is low, the potential clinical impact may be significant. In a pacemaker-dependent patient, there is a possibility that EMI from a cellular phone may lead to inhibition of pacing that can result in syncope [12]. EMI may also lead to asynchronous pacing which can cause symptoms or in an extremely unlikely scenario may lead to ventricular arrhythmia from R-on-T phenomenon. There is potential for erroneous atrial or ventricular sensing leading to inappropriate ICD therapies or mode switching [13]. In extremely rare cases, EMI may cause power-on reset in CIEDs although this is unlikely from cellular phone RF energy [14]. Current generation devices employ noise detection algorithms which has decreased the potential clinical impact of this type of interaction.

## Cellular Phone Magnets and CIED Interactions

CIEDs are intentionally designed to respond to external static magnetic fields via magnetic reed switches, Hall-effect sensors, giant magnetosensitive resistors, or telemetry coils [15]. The reed switch was commonly used in older generation CIEDs and consists of two metal strips in a glass capsule. When exposed to a magnet, these metal strips come into contact and lead to a change in electric potential difference that is sensed by an amplifier. A Hall-effect sensor utilized in MRI conditional devices works by generation of electric potential difference across a conductor when a magnetic field is perpendicular to the direction of current flow. This has more predictable behavior compared with a reed switch and has the ability to “lockout” when undergoing an MRI [16]. Giant magnetosensitive resistors were introduced in Abbott devices and consist of thin layers of ferro-magnetic and non-magnetic materials. When exposed to a static magnetic field, these layers line up in a way that lowers electrical resistance and activates designated electronic switches on the implanted device. The magnetic field strength required to induce magnet mode varies from device manufacturer and the type of magnetic switch used on the specific device. A magnetic strength as low as 10 gauss has the capability to induce magnet response in select devices [17].

Neodymium-iron-boron (neodymium) magnets are rare earth magnets that were initially discovered in the 1980s [18]. These are small, strong, inexpensive magnets that are increasingly being incorporated in electronics, toys, jewelry, smartphones, smartwatches, headphones, and E-cigarettes. Unlike traditional magnets, a patient may not always be aware of being exposed to neodymium magnets since they are often small and may be hidden. Multiple studies have found that they have the potential to cause magnet interference on CIEDs at close distances [19, 20].

Recent generation of smartphones have started to incorporate increasing amounts of neodymium magnets for optimization of wireless inductive charging and attachment of accessories. During wireless inductive charging, a current is passed through a coil in the charger. This in turn creates an electromagnetic field that the receiving plate on the smartphone comes in contact with which generates a current that is used to charge the smartphone. The Wireless Power Consortium is an international organization that has standardized wireless inductive charging into what is termed the “Qi” standard. Apple, Samsung, and Xiaomi are three of the biggest smartphone manufacturers and all are part of the Wireless Power Consortium [21]. Samsung adapted wireless inductive charging in 2015 on the Samsung Galaxy S6 and S6 Edge devices and Apple in 2017 with the iPhone X and iPhone 8 models. On wireless “Qi” certified chargers, the Apple iPhones charge at rates up to 7.5 W [22]. Apple expanded on wireless inductive charging on the iPhone 12 series by adding a new feature called MagSafe. MagSafe is a proprietary technology which utilizes wireless inductive charging with an added magnet array for charging optimization and for attachment of accessories. It supports the wireless “Qi” standard but is able to provide inductive charging at 15 W compared to 7.5 W in the previous generations [23]. This in turn allows for faster wireless charging. The specifications and design of MagSafe are published on Apple’s website [24].

Prior to the release of Apple MagSafe technology, there were no reported cases of a smartphone causing magnet mode in CIEDs. In the previously mentioned studies by Lacour and Burri, the iPhone 6 was placed directly over the CIED and did not induce magnet mode in any of the experiments carried out [6, 9]. In a study performed by Patterson et al., the iPhone XS was tested in an ex vivo experiment and did not induce magnet mode in select CIED models [25]. In 2021, there were two reports of the iPhone 12 series with MagSafe causing magnet mode on CIEDs. Greenberg et al. demonstrated in a single in vivo experiment that iPhone 12 can induce magnet mode on a Medtronic device [26]. Nadeem et al. carried out an in vivo and ex vivo tests, demonstrating that both pacemakers and ICDs from Medtronic, Abbott, and Boston Scientific are susceptible to the magnet interference from the iPhone 12 Pro Max [27]. Following these reports, a larger scale study was carried out in 164 patients with CIEDs from all major device manufacturers. When the iPhone 12 was placed in proximity to the CIED, clinically significant magnetic interference occurred in 18.3%. The mean maximum distance from the skin surface for iPhone 12 to induce CIED magnet mode activation was 0.8 ± 1.2 mm [28••]. It is important to note that this study and the prior reports were experiments carried out by the authors rather than actual clinical scenarios with adverse patient outcomes. To our knowledge, there has yet to be a clinically significant adverse event documented to be a result of inadvertent magnet mode reversion from current generation smartphones, although this is certainly a possibility. The above-mentioned studies led to a series of media articles and a response from Apple stating that newer generation iPhones and accessories contain more magnets and have a potential for interference with medical devices. A complete list of Apple products with magnets was subsequently provided by the company [29]. The FDA updated their recommendations and reiterated that smartphones should be maintained at a distance of six inches from CIEDs [11]. A subsequent study was performed that tested the magnetic field strength of every phone in the iPhone 12 series using a FW Bell 5180 Gauss Meter. The static magnetic field strength measured was greater than 10 gauss at a distance of 1 mm to 11 mm from the surface in all devices in the iPhone 12 series, suggesting that all these phones have the potential to cause CIED to revert into magnet mode [30•].

Besides Apple iPhones, Samsung flexible display smartphones such as Galaxy Fold and Galaxy Z Flip also contain magnets that can interact with CIEDs. The main use of magnets in these phones is to keep screens folded to reduce footprint when not used. Samsung has published potential CIED interaction warnings on its website [31].

## Magnetic Interference from Smartphone Accessories

Smartphone accessories which include wireless headphones and smartwatches have also demonstrated capability of causing magnet interference on CIEDs. Headphones contain small magnets that are used to vibrate the speaker diaphragm to produce soundwaves. In a study published in 2009, 100 patients were exposed to eight different models of portable headphones. Clinically relevant magnetic interference was observed in 30% of the patients. The magnetic field strength of portable headphones was measured using a gaussmeter. In the 8 different brands of headphones tested, 7/8 (87.5%) and 2/8 (25%) demonstrated magnetic strength > 10 gauss at a distance of 1 cm and 2 cm, respectively [32]. This study was carried out in 2009 and since then portable headphone design and technology have further developed. Portable headphones are now often wireless and communicate to cellular phones via Bluetooth. Similar to smartphones, portable headphones may have the capability for wireless inductive charging. In Apple Airpods, the charging case and the individual headphones contain magnets [29]. The magnetic strength and potential for CIED interference of current generation headphones with wireless charging capability magnetic cases has not been studied to our knowledge.

**Fig. 1:**
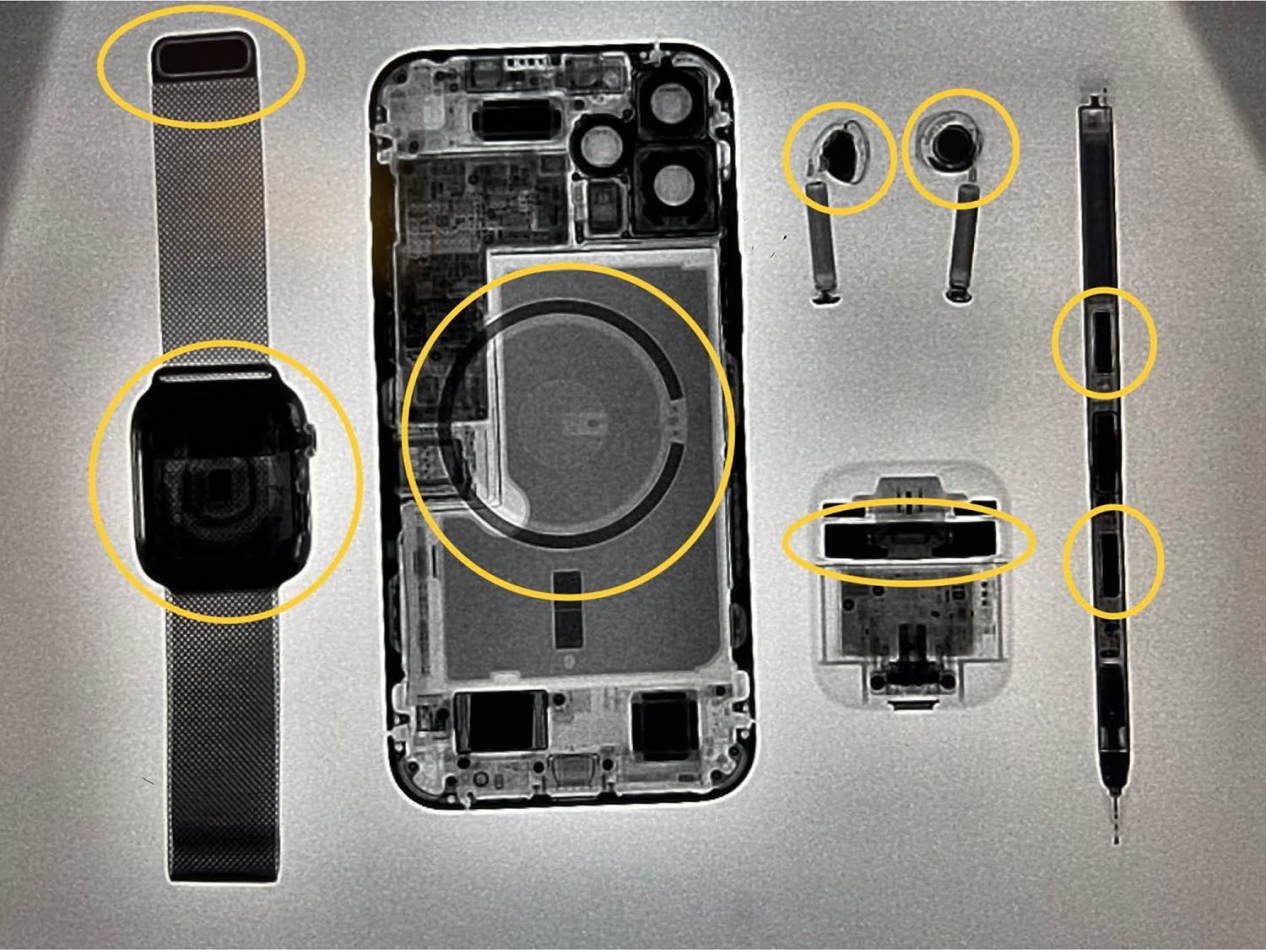
Fluoroscopic image of Apple Watch Series 5 with Milanese loop wristband, iPhone 12 Pro Max, 2nd generation Apple Airpods, and Apple Pencil 2nd generation (from right to left). The location of magnets in these devices are circled

Smartwatches are a popular smartphone accessory. In 2019, there were over 20 million smartwatches sold in the USA [33]. Smartwatches contain magnets that have the potential to cause interference on CIEDs. The static magnetic field strength of the Apple Watch Series 6 is 39.19 gauss from the back surface at a distance of 11 mm, which is greater than what is required to induce magnet mode on CIEDs [30•]. In vivo studies have been mixed on whether smartwatches can cause CIEDs to revert into magnet mode. In the study by Lacour et al., the 1st generation Apple Watch was tested and in 148 patients there was no magnet affect when the Apple Watch was placed directly over a CIED [9]. In a study testing the Apple Watch Series 3 and Samsung Galaxy Watch, a total of 684 EMI tests were carried out on 171 patients with CIEDs from five manufacturers. There were no cases of EMI under nominal or worst-case scenario programming [34]. These studies differ from the 2021 published case report where a patient presented with a complaint of ICD beeping at night. After an investigation, it was revealed that this beeping was due to magnetic reversion mode being triggered by the Apple Watch wristband. The authors subsequently tested Fitbit and the Apple Watch magnetic wristbands and found that both were able to trigger magnetic reversion mode ex vivo on a Medtronic device at distance of 2.4 and 2.0 cm, respectively [35]. While the study by Lacour and Tzeis did not demonstrate any cases of magnet reversion mode being triggered by smartwatches, the study by Seidman et al. demonstrates that the Apple Watch 6th generation has a strong enough static magnetic field to induce magnet reversion mode in CIEDs [30•]. The discrepancy among these studies may be due to the difference in the smartwatch models and the location where magnet strengths were tested. The cumulative results of these studies can be seen in Table 2. Figure 1 is a fluoroscopic image of an Apple iPhone and its accessories with the specific location of magnets encircled.

**Table 2:** Summary of cellular phone and their accessories static magnetic field and CIED interactions

**Author**	**Date of publication**	**Device**	**Number of patients and/or tests**	**Induction of magnet mode**
Burri	2016	Samsung Galaxy S4, iPhone 6	63 patients	0%
Lacour	2020	iPhone 6, Apple Watch Series 1	148 patients	0%
Asher	2020	Apple Watch Milanese loop wristband, Fitbit wristband	2 tests	100%
Nadeem	2021	iPhone 12	3 patients in vivo, 11 tests ex vivo	100% in vivo, 72.7% ex vivo
Patterson	2021	iPhone XS, iPhone 12	12 tests	33% for iPhone 12, 0% for iPhone XS
Tzeis	2021	Apple Watch Series 3, Samsung Galaxy Watch	171 patients	0%
Lacour	2021	iPhone 12	164 patients in vivo, 39 tests ex vivo	18.3% in vivo, 84.6% ex vivo

## Clinical Implications of Magnetic Interference

When exposed to a static magnetic field, ICDs and pacemakers have different responses. Pacemakers that are programmed DDD, VVI, and AAI will pace DOO, VOO, and AOO, respectively [15]. The pacing will occur at a fixed rate which differs according to the manufacturer and according to the battery status. Except for Sorin pacemakers, all other manufacturer pacemakers will revert to their original programming once the magnet is removed [15]. Biotronik, Boston Scientific, and Abbott pacemakers can be programmed to ignore external magnet response. Asynchronous pacing is generally well tolerated by most patients; however, some patients may feel symptoms due to loss of AV synchrony. In extremely rare cases, asynchronous pacing may cause life threatening ventricular arrhythmias due to R on T phenomenon [36–38].

In contrast to pacemakers, ICDs continue pacing with their programmed mode when exposed to a static magnetic field. Magnet response in ICDs disables tachytherapies. Medtronic and Boston Scientific ICDs may have an audible tone when they revert into magnet mode and Abbott devices may have a vibratory alert. Only Boston Scientific and Abbott devices can be programmed to ignore external magnets. In select Boston Scientific devices, there is a feature called “Change Tachy Mode With Magnet,” that if programmed on will disable tachytherapies even after the magnet is removed [15]. This can potentially be dangerous as a static magnet field from smartphones may permanently disable tachytherapies without the patient being aware. It is important to note that this feature is not commonly programmed on. In most current devices with current programing, the tachytherapies transiently disable and immediately re-enable once the external static magnet field is removed. The patient would only be at risk for an untreated ventricular arrhythmia while the smartphone is in close proximity to the CIED and that risk goes away after the smartphone is removed. Suspension of ICD therapy from environmental sources has been estimated to occur with an incidence of 6.9% per patient-year [39]. Although, the incidence may be relatively high, the duration of the suspension was probably short and risk of concurrent arrhythmia is probably very low. The dissemination of portable devices with strong magnets may though accentuate the risk based the increased and potentially prolonged exposures if these devices are carried in proximity to the patient’s CIEDs. At present time, to our knowledge there has been no documented case of a harmful outcome due to untreated ventricular arrhythmia from inadvertent magnet mode caused by an external static magnetic field from smartphones or their accessories. It is important to note also that if this were to occur, the device may not always record the event as detections and recordings are sometimes turned off in magnet mode depending on the manufacturer and device type. Additionally, data containing magnet reversion episodes may not always be accessible through routine interrogation unless specifically requested through company’s technical services.

## Conclusion

Smartphones, smartwatches, wireless headphones, and accessories use is growing rapidly in the USA. Newer generation of these devices are incorporating increasing amounts of magnets for various purposes such as optimization of wireless inductive charging, attachment of accessories, and convenience functionalities. These magnets have the potential to cause CIEDs to revert into magnet mode, only if in close contact and properly aligned. Magnet reversion mode in pacemakers will lead to temporary asynchronous pacing and in ICDs will transiently disable tachytherapies. If smartphones and their accessories are maintained at 6 inches distance from the CIED then the risk of inadvertent induction of magnet mode in a CIED is extremely unlikely.
